# The Influence of Occlusion Type on Oral Health-Related Quality of Life in Patients with Complete Dentures—Lingualized vs. Bilaterally Balanced Occlusion

**DOI:** 10.3390/jpm14090921

**Published:** 2024-08-29

**Authors:** Nancy Poljak, Ivan Kovačić, Nikola Petričević, Antonija Tadin, Marisa Klančnik

**Affiliations:** 1 Health Centre of Split-Dalmatia County, 21000 Split, Croatia; nancyy297@gmail.com; 2 Department of Maxillofacial Surgery, Split University Hospital, 21000 Split, Croatia; atadin@mefst.hr; 3 Department of Prosthetics, School of Medicine, University of Split, 21000 Split, Croatia; 4 Department of Removable Prosthetics, Faculty of Dentistry, University of Zagreb, 10000 Zagreb, Croatia; petricevic@sfzg.hr; 5 Department of Restorative Dentistry and Endodontics, School of Medicine, University of Split, 21000 Split, Croatia; 6 Department of Otorhinolaryngology, University Hospital Split, 21000 Split, Croatia; marisa.klancnik@st.htnet.hr

**Keywords:** lingualized occlusion, bilaterally balanced occlusion, occlusal schemes

## Abstract

Objective: This randomized, single-blind controlled study aimed to investigate the QoL and satisfaction of patients wearing complete dentures with lingualized (LO) and bilaterally balanced occlusion (BBO). Participants were stratified based on their prior experience with complete dentures. Methods: The study involved 131 complete denture wearers who were categorized into four groups: G1—first-time prosthesis wearers treated with LO (n = 33); G2—first-time prosthesis wearers treated with BBO (n = 31); G3—participants with prior prosthesis experience treated with LO (n = 34); G4—participants with prior prosthesis experience treated with BBO (n = 33). After wearing the prosthesis for one month, all participants completed the Oral Health Impact Profile (OHIP-14) questionnaire. A statistical analysis was conducted using the χ^2^ test, Kruskal–Wallis analysis and Mann–Whitney test, with significance determined at *p* < 0.05. Results: After dividing the respondents into two groups, a statistically significant difference was observed in the distribution of scores for three questions related to oral pain severity, anxiety, and difficulty relaxing. However, the distribution of scores for all other questions did not show a statistically significant difference between the groups studied (*p* < 0.05). The total OHIP-14 score was also not statistically different (z = 0.469; *p* = 0.639). However, when respondents were divided into four groups, the median score for first-time denture wearers was 3.9 points higher in respondents who received dentures with BBO compared to those with LO (*p* < 0.001). Furthermore, the median score for first-time denture wearers who received BBO was higher than for those in the second group who received BBO (*p* = 0.013). Conclusion: Patients wearing complete dentures for the first time demonstrated significantly higher satisfaction with the LO scheme compared to the BBO scheme. In contrast, satisfaction levels between occlusal schemes did not significantly differ among patients with prior denture-wearing experience. Novice denture wearers reported heightened sensations of oral discomfort, anxiety, and difficulty relaxing regardless of the occlusal scheme compared to experienced wearers, likely due to the unrealistic expectations that first-time wearers often have about complete dentures.

## 1. Introduction

The World Health Organization (WHO) defines health broadly as “a state of complete physical, mental and social well-being and not merely the absence of disease or infirmity”. This comprehensive definition of health extends to both general and oral health [1]. While tooth loss itself is not classified as a disease, its cumulative effects over time often affect oral function, self-perception, emotional balance, and overall QoL [2]. As the number of missing teeth increases, the ability to chew decreases significantly. Studies show that edentulous people have a chewing efficiency of 10–20% compared to people with complete dentition [3]. Despite advanced preventive measures, even in highly developed countries, there is a growing need for prosthetic dental treatment due to the increasing number of elderly people, resulting in a significant number of edentulous patients [4]. While dental implants are a popular alternative for completely edentulous people, the financial constraints and surgical complexity associated with implant placement ensure that complete dentures remain important in oral rehabilitation [5]. In addition to their functional role, complete dentures also have a direct impact on facial aesthetics and thus influence the psychological well-being of patients [6]. The success of dentures depends on a variety of factors, including the anatomical properties of soft and hard tissues, the condition of the oral mucosa, the presence or absence of xerostomia, the psychological state of the patient, and the occlusal scheme, which affects stability, retention, the distribution of occlusal forces, aesthetics, chewing function, patient comfort, and overall satisfaction with the prosthesis [7]. While there are various objective criteria for evaluating dentures, such as counting the number of sore spots, measuring EMG activity, or assessing chewing efficiency, it is also important to gather direct feedback from patients. Using patient-reported outcome measures can provide valuable insights into how well patients adapt to and perceive their dentures [8].

Complete dentures have different biomechanical properties compared to natural dentures. With them, the force acting on one tooth is transferred to the entire denture. To control the lateral forces that can destabilize the prosthesis, teeth with different morphologies and different occlusion schemes have been developed [9,10]. Unfavorable muscle forces during function result in undesired movements of the prosthesis. One way to minimize this is to use multiple contacts on the working and non-working side [11]. The number and strength of the occlusal contacts determine the magnitude and direction of the forces that are transferred to the residual ridges via the denture base [12]. A correct occlusal scheme enhances QoL, reduces the complexity of denture fabrication, and simplifies the process of occlusal adjustment [13]. Several clinicians and patients believe that the success of dental treatments could be affected by the dentist’s experience; however, the results on this issue are inconclusive [14]. Occlusal schemes are diversified and continuously change over time, and hence, today’s restorative dentists are in a state of uncertainty as to which occlusal schemes can be satisfactorily incorporated into dentures [15].

BBO involves simultaneous contacts of the teeth of both jaws on both sides across the anterior and posterior occlusal area during centric relation and eccentric mandibular positions as well as during lateral and protrusive mandibular movements [10]. BBO is considered a good occlusal scheme as it provides excellent stability and retention and facilitates the adaptation of the prosthesis. In contrast, in LO, there is primarily contact between the palatal cusps of the upper molars and premolars and the enlarged central fissure of the lower molars, while the buccal cusps of the upper posterior teeth are disoccluded [9,16]. LO is often praised for its aesthetics, ease of fitting, and excellent retention and stabilization during masticatory function [16].

According to the research conducted by Garry Goldstein et al. [17] there is strong support that the average denture patient, with good residual ridges and no neuromuscular problems, will function adequately with a properly fabricated complete denture, regardless of the occlusal scheme. In a study conducted by Kawai Y. et al., the initial observations after the insertion of complete dentures showed no significant difference between LO and BBO in terms of patient satisfaction. However, a substantial improvement in patient satisfaction with LO dentures was observed over a six-month period, especially in individuals with highly resorbed mandibular ridges, where a remarkable improvement in retention, overall satisfaction, and QoL was observed [18]. Another study by Ramya et al. [19], which used the Oral Health Impact Profile and the Visual Analog Scale to measure the satisfaction and QoL of patients who received either BB or non-balanced occlusal concepts, found no statistically significant difference in satisfaction between the users of these two occlusal schemes. Several authors have conducted various meta-analyses to determine whether one occlusal scheme is superior to another [20].

Given the different characteristics of BBO and LO in complete dentures, this study aims to investigate potential differences in QoL related to oral health between users of these two occlusal concepts, with a focus on their prior experience with dentures. The research hypothesis states that there is no statistically significant difference in patient satisfaction regarding oral health between individuals receiving BBO and LO regimens in both respondent groups (regardless of whether they have previous denture experience or not).

## 2. Materials and Methods

The study involved completely edentulous patients who visited the prosthetic department at the Dental Polyclinic in Split, Croatia, from 15 October 2019, to 1 March 2023, seeking new full dentures. The research was conducted in accordance with the Declaration of Helsinki and received approval from the Ethics Committee of the Dental Polyclinic in Split on 29 November 2019.

A total of 131 patients (68 women and 63 men) aged 56 to 83 years, with an average age of 66 years, were included in the study. This randomized clinical trial was single-blind; i.e., the patients were unaware of which occlusal concept was used in their full denture. However, the clinician was informed of the assigned occlusion type. All patients signed a consent form, and there was no financial support for the study other than patients using their health insurance to cover the cost of the dentures.

Inclusion criteria included individuals of all genders who required new dentures due to reasons such as fractures, misplaced dentures, and functional and aesthetic limitations of existing dentures, as well as first-time denture wearers. Exclusion criteria included individuals with recent extractions, acute disease, TMJ problems, uncooperativeness, masticatory dysfunction, inability to complete the OHIP-14 questionnaire, xerostomia, and severe oral manifestations of systemic disease. For the data analysis, PCA was conducted, yielding a KMO measure of 0.794, Bartlett’s test of sphericity of χ^2^ = 620.3 (df = 91; *p* < 0.001), and Cronbach’s alpha of 0.850 (with alpha values for deleted items ranging from 0.834 to 0.847). Patients were divided into four groups based on their denture-wearing experience and their dependence on the concept of occlusion in complete dentures, using a block randomization procedure via a computer software program (Mahmood Saghaei Allocation Software, Isfahan, Iran) [21]. First, participants were split into two main groups: those who wore complete dentures for the first time and those who had previous experience of wearing dentures. Subsequently, each main group was further divided into two additional subgroups based on the use of two different occlusion schemes: LO and BBO. At the beginning of the study, complete maxillary and mandibular dentures were fabricated for all participants according to standardized criteria. An anatomical impression was taken with irreversible hydrocolloid alginate (Aroma Fine Plus, GC Corporation, Tokyo, Japan) using a factory tray. A functional impression was then taken using a tray fabricated on the basis of the anatomical model, using thermoplastic material (Greenstick, KerrHawe SA, Bioggio, Switzerland) for the functional margins and condensation silicone (Xantopren, Heraeus–Kulzer, Hanau, Germany) for the overall impression. An adaptable SAM 3 articulator (SAM Prazisionstechnik GmbH) with appropriately mounted semi-anatomical acrylic teeth (SR Vivodent PE, Ivoclar Vivadent, Schaan, Liechtenstein) was used to replicate the interocclusal relationships and jaw movements. All clinical procedures were performed by the same prosthodontist, while an experienced technician oversaw the laboratory aspects. In the fabrication of dentures with LO, the shape and positioning of the teeth were altered compared to those using BBO. In the LO scheme, the upper posterior teeth were rotated slightly buccally so that only their palatal cusps came into contact with the central fissure of the lower teeth, while the buccal cusps were excluded from both centric relation and eccentric mandibular movements. In addition, the central fissure of the lower posterior teeth was widened. In contrast, the BBO dentures were configured in such a way that the anterior and posterior teeth came into contact with their antagonists simultaneously in centric relation and during eccentric movements. The contacts on the balanced side were between the maxillary palatal cusps and the mandibular buccal cusps, with the maxillary palatal cusp touching the central fissure of the mandibular teeth, while the mandibular buccal cusp came into contact with the central fissure of the maxillary teeth. QoL was assessed using the OHIP-14 questionnaire, which was translated from English to Croatian [22]. At a check-up visit one month after receiving their complete dentures, patients were provided with a paper copy of the OHIP-14 questionnaire by the prosthodontist who had fitted their dentures (they were instructed to complete the questionnaire independently at home). The OHIP-14 questionnaire consists of 14 questions about the impact of oral health on their QoL, specifically concerning their new complete dentures. The responses were rated on a five-point scale ranging from 0 (never) to 4 (very often). Consequently, each question’s score ranged from 0 to 4, which contributed to the total score when all questions were added together. A higher total score indicates a higher level of patient dissatisfaction. One patient was excluded from the study due to incomplete responses. The data were analyzed using the Statistical Package for the Social Sciences, Version 26 (SPSS, IBM Corp, Armonk, NY, USA). The Kolmogorov–Smirnov test was used to check the normality of the response distribution. A descriptive analysis was performed to determine the frequency and percentage of categorical data, while quantitative data were expressed as means with the corresponding standard deviations or medians along with the interquartile ranges. In the data analysis, we used the χ^2^ test for contingency tables. A Kruskal–Wallis analysis and Mann–Whitney test were applied to compare the quantitative data among and between the analyzed groups. The standardized effect size was expressed by η^2^ for Kruskal–Wallis (χ^2^/(n − 1)). The standardized effect size for the Mann–Whitney test was r (r = Z/sqrt(n)). The results were interpreted at the significance level of *p* < 0.05.

## 3. Results

The study comprised two groups: one group of subjects wearing a total prosthesis for the first time (n = 64) and another group of subjects who had received a complete denture previously (the second time or more) (n = 67). These two groups were comparable in terms of gender (χ^2^ = 0.006; *p* = 0.938) and occlusion type (χ^2^ = 0.009; *p* = 0.926). However, the group with previous denture experience was slightly older than the first-time denture group (z = 2.0; *p* = 0.045). This age difference is not considered clinically significant (Table 1).

All subjects completed the OHIP-14 questionnaire, which consists of 14 questions. The distribution of respondents according to the number of points for each individual question is shown in Table 2. The analysis revealed no statistically significant differences between the groups for the following questions: “Did or do you have difficulty pronouncing words?”, “Did or do you have an unpleasant taste?”, “Were or are you uncomfortable eating a certain type of food?”, “Did you think about your teeth, mouth, jaw, or prosthetic work?”, “Do you think your diet is unsatisfactory?”, “Did you have to interrupt a meal?”, “Have you felt unwell?”, “Have you been irritable towards others?”, “Have you had problems completing everyday tasks?”, “Have you ever felt that you were not functioning at all?”, and “Do you feel that life offers you less satisfaction?” (*p* > 0.05). Due to the small number of subjects who responded with 2 or 3 points to questions 2, 7, and 8, these scores were combined into a single group for analysis. Overall, the total score on the OHIP-14 did not show a statistically significant difference between the groups (z = 0.469; *p* = 0.639).

The distribution of respondents’ answers to the question “Did you have or do you have severe pain in your mouth?” (question 3) showed a statistically significant difference at the 94% significance level between the groups studied (χ^2^ = 5.58; *p* = 0.061). The difference results from the number of respondents who combined the answers “sometimes” and “often”. In the group of respondents who received a full denture for the first time, five (7.8%) respondents chose these answers, while none in the group receiving a full denture for the second time or more selected them (Table 3). Similarly, the distribution of responses to the question “Are you afraid because of problems with your teeth, mouth, jaws, or prosthetic work?” (question 6) differed statistically significantly between the groups studied. This difference is due to the number of respondents who answered “sometimes” (2 points), which is represented by eight (12.5%) respondents in the group receiving a full prosthesis for the first time and only one (1.5%) respondent in the group receiving a full prosthesis for the second time or more (Table 3). The distribution of respondents to the question “Do you find it harder to relax because you have problems with your teeth, mouth, jaws, or prosthetic work?” (question 9) showed a statistically significant difference between the groups studied. The difference is caused by the “never” (0 points) response, with 8 (12.5%) of participants in the group receiving a full denture for the first time choosing this answer, compared to 4 (6%) in the group receiving a full denture for the second time or more. Additionally, the difference was also influenced by the “sometimes” (2 points) response. None of the respondents in the group receiving a full denture for the second time or more selected this option (Table 3).

For further analysis, the participants were categorized into four groups (Table 4):

- Group of subjects receiving a full denture for the first time with an LO (n = 33);
- Group of subjects receiving a full denture for the first time with a BBO (n = 31);
- Group of subjects receiving a full denture for the second time or more with an LO (n = 34);
- Group of subjects receiving a full denture for the second time or more with a BBO (n = 33).

We examined whether there were differences in the total OHIP-14 scores across these four groups. The Kruskal–Wallis analysis showed no statistically significant difference in subject age among the four groups (χ^2^ = 4.2; *p* = 0.235). However, the Kruskal–Wallis analysis of the sum of the total OHIP-14 scores showed a statistically significant difference between the four groups (χ^2^ = 20.1; *p* < 0.001) (standardized effect size η^2^ = 0.15). Within the group participants with LO, the median QoL score was higher by one (1) for those receiving a full denture for the second time or more compared to those receiving it for the first time (z = 2.47; *p* = 0.013; standardized effect size r = 0.30). Within the group participants with BBO, the median of QoL was higher by three (3) for those receiving a full denture for the first time compared to those receiving it for the second time or more (z = 3.35; *p* = 0.001; standardized effect size r = 0.41). However, there was no statistically significant difference in QoL between participants receiving a full denture for the first time and those receiving it for the second time or more (z = 0.469; *p* = 0.639).

## 4. Discussion

This study indicates that overall patient satisfaction does not differ significantly between the groups for most items on the questionnaire. However, significant differences were observed in specific areas: individuals who received dentures for the first time reported greater issues with mouth pain, anxiety, and relaxation difficulties compared to those who had previous denture experience. This observation can be explained by the fact that patients with prior denture experience generally adapt more easily and quickly to new dentures.

A finding from the study that partially contradicts the original hypothesis is that patients in the group wearing full dentures for the first time expressed statistically significantly higher satisfaction with the LO scheme compared to the BBO scheme, according to the total questionnaire scores. This result is remarkable as no previous study comparing occlusal concepts divided the participants into these two specific groups mentioned and reached this conclusion. In contrast, for patients with prior denture experience, no significant difference was found between the LO and BBO occlusion schemes. Furthermore, among patients who received BBO, those receiving their first prosthesis reported lower satisfaction compared to those who had already used at least one full denture. This suggests that individuals with previous denture experience tend to adapt more favorably to the BBO scheme than those using it for the first time.

Although the mechanism that makes LO in heavily resorbed ridges more satisfactory than other types of occlusion is complex to explain, the most important factor is the reduction in lateral destabilizing forces, which makes the prosthesis more stable and thus chewing more comfortable and easier for the patient [18]. Yuichi Matsumaru [23] also confirmed in his study that patients with a large resorption of the lower alveolar ridge were more satisfied with the LO as the occlusal scheme. However, considering that more resorbed ridges are associated with years of edentulism and, therefore, a greater number of dentures fabricated during this time, one would expect patients who already have experience with two or more dentures to be more satisfied than the group of completely edentulous patients receiving a denture for the first time. The results of the study by A. F. Sutton et al. [24] show that patients receiving a full denture for the first time are more satisfied with the LO scheme. This is consistent with the results of our study and can be explained by the fact that the forces that have a stabilizing effect on full dentures are transferred better, i.e., to the base of the upper and lower full denture, so that it can be expected that patients receiving a full denture for the first time are more satisfied with LO than with BBO. Savvas N. discovered that patient adaptation to and satisfaction with newly constructed dentures improved significantly for both BBO and LO denture groups throughout the observation period, i.e., 3 and 6 months after the prostheses were delivered [25].

In the study conducted by Jiyar Amin Ali and Rizgar Mohammed Ameen Hasan [26], the ability and ease of chewing nuts with LO and BBO prostheses were measured. The pieces of nuts crushed by chewing by individuals with LO on full dentures were uniform and of similar size, as opposed to irregular or different-sized pieces of nuts in BBO wearers on full dentures. The reason for this is the better stability of the dentures during chewing in LO wearers, whereas patients with BBO complained of difficulty and pain during chewing due to denture movement during chewing. The stability achieved by LO occurs because the palatal cusps of the upper posterior teeth transmit forces directly to the center of the lower teeth, which automatically prevents movement of the lower denture during mastication while avoiding lateral rotational forces that create double contacts in the posterior teeth in BBO. Neelam A Salvi et al. [20] compared the results of 12 different studies in their meta-analysis. In their studies, various authors used different tests to assess patient satisfaction, such as OHIP-EDENT, OHIP-20, CDS, DSQ, GOHAI, DPSQ, and VAS questionnaires, and concluded that the majority of the studies considered gave preference to LO over BBO in terms of greater patient satisfaction and chewing ability. Umair Wali Khan et al. [27] also confirmed in their study that the efficiency of chewing is better in carriers of the LO scheme than in carriers of the BBO scheme. They came to this conclusion by examining the weight of the chewed peanuts that the patients had to chew until the chewed bolus was ready to swallow and then spit it out in a sieve. Una Sobelova and Irena Rogovska [28], on the other hand, concluded that overall patient satisfaction with dentures depends on the degree of comfort when wearing and using the two full dentures, which is mainly related to their stability and retention, but also that the aesthetic factor plays an important role in making patients feel comfortable wearing their prosthetic restorations.

Choosing a suitable occlusion scheme for complete dentures is always a major challenge [29]. When choosing the type of occlusion for edentulous patients, the functional needs and psychological factors must be taken into account [30]. Different types of occlusion of complete dentures have been investigated by many authors over the long term, and numerous studies have considered and compared different occlusion schemes depending on certain parameters [31,32]. C.A.A. Lemos et al. concluded that although BBO is widely used, it does not perform significantly better than other regimens in terms of patient satisfaction, whereas LO has shown consistent efficacy in terms of overall satisfaction and chewing ability [5]. Similarly, J. Patel et al. point out that although the differences between the schemes may not be significant, BBO can still be considered the optimal occlusal scheme [13]. Caitlin Grech et al. [33] also reported that the choice of occlusal scheme did not significantly affect patient-reported subjective outcomes. To date, there is no definitive conclusion as to which type of occlusion scheme is best suited for the fabrication of functional complete dentures and with which type patients would be most satisfied [34].

When considering the entire study population, the results are consistent with a large number of studies that also failed to prove that one occlusal concept is better than another. Certainly, other factors should also be considered when evaluating patient satisfaction with their prosthetic replacement, such as the patient’s positive or negative attitude towards the therapy and the patient’s expectations of the therapy and then the experience with previous prosthetic restorations, but also the relationship between the patient and the therapist as well as the patient’s confidence and their assessment of the therapist’s knowledge and ability to perform the therapy well.

One limitation of this study is the small sample size, which makes the study less meaningful. In order to obtain more objective and precise conclusions or results, the number of participants should be increased. Another limitation is that this was a single-blind study. A double-blind study with a larger number of subjects would increase the validity of some studies though this was not feasible within the scope of this study.

## 5. Conclusions

Based on the study’s findings, the following conclusions can be drawn:

1. Patients wearing full dentures for the first time show significantly higher satisfaction with the LO scheme compared to the BBO scheme, while there is no difference in no significant difference in satisfaction between the two occlusal schemes among patients with prior experience with full dentures.
2. A comparison between patients from both groups who have received BBO on their dentures shows that patients wearing dentures for the first time report lower satisfaction compared to those who have had dentures previously.
3. The study reveals that first-time denture wearers experience more significant issues with mouth pain, anxiety, and relaxation difficulties compared to those who have worn dentures before, regardless of the occlusion scheme received, which can be linked to the fact that those patients who receive dentures for the first time often have unrealistic expectations and therefore encounter a greater number of problems related to these dentures.

## Figures and Tables

**Table 1:** Distribution (%) of subjects according to the qualitative variables examined and the median; minimum and maximum age of subjects.

		Patients with Full Dentures		
		First Time	Second Time or More	*p*
Occlusion	Bilaterally balanced	31 (48)	33 (49)	0.926 *
	Lingualized	33 (52)	34 (51)	
Gender	Female	33 (52)	35 (52)	0.938 *
	Male	31 (48)	32 (48)	
Age (years)		69; 56–78	70; 60–83	0.056 **

Data are presented as numbers (percentages) and median (interquartile range). *p* < 0.05; * χ^2^ test, ** Mann–Whitney test.

**Table 2:** Relationship between selected OHIP-14 questions and the studied groups.

		Group		
	Number of Points	Patients Receiving Dentures for the First Time (n = 64)	Patients Receiving Dentures for the Second Time or More (n = 67)	*p*
Have you had or do you have difficulties pronouncing words?	0	25 (39.1)	26 (38.8)	0.109 *
	1	35 (54.7)	41 (61.2)	
	2	4 (6.2)	0 (0)	
Have you felt or do you feel an unpleasant taste? (P2)	0	30 (46.9)	39 (58.2)	0.119 *
	1	31 (48.4)	28 (41.8)	
	2	2 (3.1)	0 (0)	
	3	1 (1.6)	0 (0)	
Have you had or do you feel discomfort when eating certain types of food? (P4)	0	7 (10.9)	5 (7.5)	0.261 *
	1	55 (85.9)	62 (92.5)	
	2	2 (3.1)	0 (0)	
Have you thought about your teeth, mouth, jaw, or dentures? (P5)	0	15 (23.4)	14 (20.9)	0.099 *
	1	45 (70.3)	53 (79.1)	
	2	4 (6.2)	0 (0)	
Do you think your diet is unsatisfactory? (P7)	0	4 (6.2)	3 (4.5)	0.472 *
	1	54 (84.4)	61 (91)	
	2	5 (7.8)	3 (4.5)	
	3	1 (1.6)	0 (0)	
Have you had to interrupt a meal? (P8)	0	22 (34.4)	24 (35.8)	0.2 *
	1	39 (60.9)	43 (64.2)	
	2	2 (3.1)	0 (0)	
	3	1 (1.6)	0 (0)	
Have you felt even a little uncomfortable? (P10)	0	18 (28.1)	26 (38.8)	0.276 *
	1	45 (70.3)	41 (61.2)	
	2	1 (1.6)	0 (0)	
Have you been irritable towards others? (P11)	0	27 (42.2)	27 (40.3)	0.565 *
	1	36 (56.2)	40 (59.7)	
	2	1 (1.6)	0 (0)	
Have you had trouble with everyday tasks? (P12)	0	28 (43.8)	29 (43.3)	0.585 *
	1	35 (54.7)	38 (56.7)	
	2	1 (1.6)	0 (0)	
Have you ever failed to function at all? (P13)	0	37 (57.8)	44 (65.7)	0.062 *
	1	22 (34.4)	23 (34.3)	
	2	5 (7.8)	0 (0)	
Do you feel that your life offers less satisfaction? (P14)	0	32 (50)	37 (55.2)	0.113 *
	1	28 (43.8)	30 (44.8)	
	2	4 (6.2)	0 (0)	
Total number of points		9.5; 4–32	9; 4–15	0.639 **

Data are presented as numbers (percentages) and median (interquartile range). * χ^2^ test, ** Mann–Whitney test; *p* < 0.05. Note: a score of 0 means “never”; a score of 1 means “almost never”; a score of 2 means “sometimes”; a score of 3 means “often”.

**Table 3:** The number (%) of respondents according to their answers to questions P3, P6, and P9.

		Group		
	Scores	Patients Receiving Dentures for the First Time (n = 64)	Patients Receiving Dentures for the Second Time or More (n = 67)	*p*
Have you had or do you have severe mouth pain? (P3)	0	5 (7.8)	7 (10.4)	0.061 *
	1	54 (84.4)	60 (89.6)	
	2	4 (6.2)	0 (0)	
	3	1 (1.6)	0 (0)	
Do you feel anxious? (P6)	0	11 (17.2)	13 (19.4)	0.045 *
	1	45 (70.3)	53 (79.1)	
	2	8 (12.5)	1 (1.5)	
Do you find it harder to relax? (P9)	0	8 (12.5)	4 (6)	0.042 *
	1	52 (81.2)	63 (94)	
	2	4 (6.2)	0 (0)	

Data are presented as numbers (percentages). * χ^2^; *p* < 0.05. Note: a score of 0 means “never”; a score of 1 means “almost never”; a score of 2 means “sometimes”; a score of 3 means “often”.

**Table 4:** Association between total OHIP 14 scores and different participant groups.

Groups	Total OHIP 14 Score	*p*
	Median; min–max	
Four groups of participants:		<0.001 *
Participants receiving full prosthesis for the first time		
LO (n = 33)	8; 5–25	
BBO (n = 31)	12; 4–32	
Participants receiving full prosthesis for the second time or more		
LO (n = 34)	9; 5–15	
BBO (n = 33)	9; 4–14	
Two groups of participants with LO:		0.013 **
Participants receiving full prosthesis for the first time n = 33	8; 5–25	
Participants receiving full prosthesis for the second time or more n = 34	9; 5–15	
Two groups of participants with BBO:		0.001 **
Participants receiving full prosthesis for the first time n = 31	12; 4–32	
Participants receiving full prosthesis for the second time or more n = 33	9; 4–14	
Intergroup		0.639 **
Participants receiving full prosthesis (LO or BBO) for the first time n = 64	9.5; 4–32	
Participants receiving full prosthesis (LO or BBO) for the second time or more n = 67	9; 4–15	

* Kruskal–Wallis test; ** Mann–Whitney test.

## Data Availability

Data are available upon request from the corresponding author.
